# Recent Insights into the Physio-Biochemical and Molecular Mechanisms of Low Temperature Stress in Tomato

**DOI:** 10.3390/plants13192715

**Published:** 2024-09-28

**Authors:** Kwanuk Lee, Hunseung Kang

**Affiliations:** 1 Department of Biology, Jeju National University, Jeju 63243, Republic of Korea; 2 Department of Applied Biology, College of Agriculture and Life Sciences, Chonnam National University, Gwangju 61186, Republic of Korea

**Keywords:** cold stress, morphological trait, C-repeat binding factor (CBF), RNA and DNA methylation, epigenetic regulation, climate change

## Abstract

Climate change has emerged as a crucial global issue that significantly threatens the survival of plants. In particular, low temperature (LT) is one of the critical environmental factors that influence plant morphological, physiological, and biochemical changes during both the vegetative and reproductive growth stages. LT, including abrupt drops in temperature, as well as winter conditions, can cause detrimental effects on the growth and development of tomato plants, ranging from sowing, transplanting, truss appearance, flowering, fertilization, flowering, fruit ripening, and yields. Therefore, it is imperative to understand the comprehensive mechanisms underlying the adaptation and acclimation of tomato plants to LT, from the morphological changes to the molecular levels. In this review, we discuss the previous and current knowledge of morphological, physiological, and biochemical changes, which contain vegetative and reproductive parameters involving the leaf length (LL), plant height (PH) stem diameter (SD), fruit set (FS), fruit ripening (FS), and fruit yield (FY), as well as photosynthetic parameters, cell membrane stability, osmolytes, and ROS homeostasis via antioxidants scavenging systems during LT stress in tomato plants. Moreover, we highlight recent advances in the understanding of molecular mechanisms, including LT perception, signaling transduction, gene regulation, and fruit ripening and epigenetic regulation. The comprehensive understanding of LT response provides a solid basis to develop the LT-resistant varieties for sustainable tomato production under the ever-changing temperature fluctuations.

## 1. Introduction

The tomato, a member of the *Solanaceae* family, is a sessile plant and one of the most crucial vegetable for maintaining modern human health and food security [1]. Tomato fruits are highly rich in nutritional compounds, including abundant vitamins and essential minerals, as well as beneficial substances, containing fibers, phenolic compounds, and lycopene, which are routinely utilized both fresh and as essential ingredients in many cuisines such as sauces, salads, and juices [2,3,4]. Tomatoes, in particular, rank as the second most important vegetable in the world and their global importance has been steadily rising owing to their dietary and commercial value [1,3]. Since its introduction to Europe in the 16th century, it has been cultivated in a broad spectrum of climate conditions, ranging from tropical to temperate [5]. However, the current abrupt climate changes including low and high temperatures have limited the growth, development, and cultivation of tomatoes in the world, thus challenging their yield and production [6,7,8,9,10].

LT stress influences tomato morphology, such as leaf structure, truss appearance, plant height, flowering, fruit development, and fruit ripening [11,12]. Also, LT is involved in physiological and biochemical aspects, including chlorophyll content, various photosynthetic parameters, membrane stability, osmolytes and polyamines (PAs) regulation, and ROS production [13,14]. Although the relationships among traits and factors-involving LT tolerance at individual stage have been extensively studied, the correlation between the evaluated traits and factors in the response of tomato to LT stress remains to be investigated [12,13,15]. Furthermore, several recent papers have described the understanding of the physiological and biochemical mechanisms at molecular levels in response of diverse crops to LT. In tomato plants, it is still lack of sufficient information to understand regulatory networks of the key modulators via CBF-dependent and CBF-independent pathways as well as m^6^A RNA methylation and epigenetic factors (DNA methylation and histone modifications) in fruit ripening and LT stress [16,17,18]. In this review, we first discuss the recent comprehensive understanding of morphological, physiological, and biochemical status, including a variety of photosynthetic parameters, osmolytes (prolines, soluble sugars, and glycine betaine), polyamines (PAs), ROS generation, and antioxidant pathways with current functional genomic studies in the response of tomato to LT stress. We also describe the previous and current understanding molecular processes—from the perception and response, the signaling cascades related to Ca^2+^, ROS molecules, CBF-dependent and -independent pathways, and epigenetic modification—to the cellular responses of cold-responsive genes (CORs) during LT stress, which will be crucial for accelerating tomato breeding program and enhancing LT tomato tolerance by enabling the early selection of LT-tolerant tomato plants with high fruit yields.

## 2. Morphological Changes of Tomato Plants in Response to LT

Low temperature (LT) is crucial for tomato plants growth and development during vegetative and reproductive growth stages. LT stress leads to delayed seed germination and poor germination rate [11,19] and LT significantly influences the leaf morphology including leaf length (LL) and leaf width (LW), truss appearance, plant height, and stem diameter (SD) in tomato plants, resulting in retarded vegetative growth [13,20]. Moreover, LT is crucial for reproductive index including pollen and ovule development, the number of flower (NFR) and fruit (NF), fruit set (FS), fruit ripening, and fruit yield (FY), which are considered as the most important and vulnerable factors affecting the tomato yields and production during LT stress [15,21,22]. Notably, the previous and recent efforts for understanding a correlation of vegetative and reproductive traits shows that the FY is positively correlated with NFR and FS [12,19,23]. Although the seed germination and vegetative growth factors are not significantly correlated, LL parameter is strongly correlated with LW and SD of vegetative parameters, indicating that vegetative parameters in LT are not highly correlated with reproductive parameters and each parameter needs to be evaluated during the different stages for selecting high yielding tomato cultivars under LT [12,19]. However, it is still lack of sufficient knowledge to dissect the close relationship between vegetative and reproductive parameters.

## 3. Physiological and Biochemical Changes of Tomato Plants to LT

### 3.1. Chlorophyll Contents and Photosynthetic Parameters

LT plays an important role in the chlorophyll biogenesis in plants and several studies have been determined in the reduction of chlorophyll contents in LT-sensitive tomato plants during short term condition [15,22,24]. The overexpression of SiFBA5 enhances cold tolerance with increment in chlorophyll contents [25]. However, the chlorophyll content of many tomato plants increases continuously during the growth and developmental stage in long term LT conditions of the greenhouse [12,13]. Even the chlorophyll levels are higher in LT than in optimal conditions. In addition, photosynthetic parameters, including F*v*/F*m*, net photosynthetic rate (*P_N_*), intercellular CO_2_ concentration (*Ci*), transpiration rate (*Tr*) and stomatal conductance (*Gs*) significantly declined in LT-sensitive tomatoes, whereas the parameters increased in LT-tolerant one compared to those in optimal condition [24,25,26]. Although the photosynthetic parameters are shown to be decreased in the early period of LT treatment, no distinct pattern or difference was observed in between the LT and control conditions in a late period of LT condition [22]. These results suggest that these indicators should be carefully applied for using criteria for selecting LT-tolerant tomato plants depending on growth and developmental stages.

### 3.2. Cell Membrane and Relative Electrolyte Leakage (REL)

Cellular membranes are mainly composed of phospholipid bilayers and play crucial roles in transport, maintenance of cell structure, cell to cell recognition, and cell signaling. Proper membrane functions allow plants to respond and adapt to environmental changes such as low temperature, high temperature, salinity, and drought by adjusting membrane lipid composition [27,28,29]. In particular, LT stress affects the saturation/unsaturation ratio of fatty acids and the protein/lipid structure in the plasma membrane, resulting in changes of the membrane fluidity and membrane stability, thus resulting in an increase in electrolyte leakage (REL) [24,30,31]. Recent several studies have reported that LT-sensitive tomato plants increase in REL levels under LT conditions compared to those in WT and LT-tolerant plants. For instance, overexpression of tomato LeGPA1, LeCOR413PM2, and ShPP2-1 which confer cold tolerance decreases REL levels in the response of the tomato to LT, whereas the REL is remarkably promoted in the RNA interference transgenic lines (RI) of LeGPA1 and LeCOR413PM2 [11,32,33]. Moreover, the SlREC2-silenced tomato plant was shown in an increment of REL level under LT stress compared to that in wild-type [26]. These results suggest that plants preferentially protect the integrity of the plasma membrane, which will be a primary target of damage, and regulate the lipid composition of the membrane to ensure its stability and integrity to cope with LT stress.

### 3.3. Relative Water Contents (RWC)

LT stress can impair plant’s ability to absorb water, thereby resulting in water loss and water stress. The water loss is closely associated with changes in the membrane state, which can shift from a typical fluid composition to a less fluid and semi-crystalline composition [34,35]. Leaf wilting is a prominent symptom in the response of plants to LT stress and plants exposure to LT stress are often correlated with low relative water content (RWC), which represents the plant’s ability to retain water and serves as a quantitative indicator of a plant’s water status [14,34]. Previous study has determined in a significant reduced RWC in control compared to that in acclimated tomato plants [34], whereas RWC levels were remarkably higher in salicylic acid (SA)-treated tomato plants than in control during LT treatment growth period [36]. Moreover, current genetic studies have reported that LT causes a significant reduction in RWC of RI, LeGPA1 and LeCOR413PM2 in tomato plants, compared to those in the control and the overexpressed plants [32,33]. Interestingly, hetero seedlings grafted with *Solanum habrochaites*-derived rootstocks showed the improvement of LT tolerance with increased RWC levels compared to homo seedlings grafted with *Solanum lycopersicum*-derived rootstocks [37], indicating that RWC will be an important and potential indicator of selecting LT tolerance plants for improving plant resilience to LT stress in tomato plants.

### 3.4. Proline, Soluble Sugars, and Glycine Betaine (GB)

Osmolytes play a crucial role in modulating water potential by absorbing and losing water during environmental stresses, which enable plant to maintain protein stability, turgor pressure, and membrane stability [8,38]. Proline, soluble sugar, and glycine betaine (GB) serve as osmoprotectants, ROS remover, as well as stabilizers, which are accumulated in diverse plants under LT stress in plants [8,22,39]. LT tolerance is significantly enhanced with the exogenous treatment of proline and GB prior to LT stress [40,41]. Several studies in tomato plants have clearly reported that the increment of proline contents in *Osmotin* transgenic lines enhances LT tolerance. The accumulation of proline in leaf or root with treatment of exogenous of BR and H_2_O_2_ are involved in the alleviation of LT tolerance compared to the non-treated plants [24,34,42]. In addition, several studies also demonstrated that the treatment with exogenous GB promotes tomato’s LT tolerance [43,44,45]. The expression of betaine aldehyde dehydrogenase (BADH) in OE transgenic tomato plant elevated GB levels, resulting from enhancing LT tolerance [46]. Intriguingly, recent genetic studies have shown that the high levels of proline, soluble sugars, and GB are observed in *high pigment 1* mutant compared to those in WT and *aurea* mutant in the response of tomato plants to LT stress [47]. Furthermore, the LeGPA1, LeCOR413PM2, and BOCRR1-OE tomato plants exhibited the elevated levels of proline and soluble sugar compared to those in WT or RI transgenic plants [32,33,48]. These results suggest that LT-tolerant tomato plants are closely associated with the high or rapid production of proline and soluble sugar in response to LT stress, which play a critical role in maintaining osmotic homeostasis.

### 3.5. Polyamines (PAs)

Polyamines (PAs) are a type of low molecular weight organic molecules containing multiple positively charged amino groups [49]. The PAs are key regulators of oxidative stress, nucleic acids and chromatin structure, membranes integrity, and protein activity [50,51,52,53]. Plants PAs include putrescine (Put), spermine (Spm), and spermidine (Spd) [54] and the Put is synthesized from arginine and ornithine [31]. The PAs play an essential role in the response to environmental stresses and developmental processes, including low and high temperature, drought, embryogenesis, flower and fruit development [8,55,56]. Previous studies have showed that Put synthesis is promoted in response of tomato to LT and the exogenous application of Put reduced electrolyte leakage in leaves [57]. Notably, exogenously treated Spd conferred LT tolerance to tomato seedlings via PAs metabolism as well as ROS scavenging [58]. Moreover, Put concentration was significantly higher in tolerant-tomato seedling than in sensitive-tomato seedling during the process of LT acclimation [59]. The Spd triggered nitric oxide (NO) release, which give rise to LT tolerance in tomato seedlings via the involvement of modulating antioxidant enzymes-related gene expression, including ascorbate peroxidase (APX), catalase (CAT), superoxide dismutase (SOD), and peroxidase (POD) [58,60]. Recent studies have reported that jasmonic acid-mediated Put biosynthesis via regulation of SIMYC2, a master regulator of JA signaling, alleviates LT stress in tomato plants and tomato fruit [49,61]. In addition to this, ShWRKY55 transcription factor increased the LT resistance of wild tomato by regulating ShSAMDC2 gene expression which is involved in Spd synthesis in PA metabolism [53]. Together, the increment of PAs, including Put, Spm, and Spd, are crucial for providing tomato’s LT tolerance via regulating PA synthesis-related TFs in response to LT. Further exploration is required to understand how the molecular upstream and downstream mechanisms of PAs influence proline metabolism and ROS scavenger system.

### 3.6. The ROS Generation and Regulation by Antioxidant Molecules

Reactive oxygen species (ROS) are highly reactive molecules that consist of free radical and non-radical substances, including singlet oxygen (^1^O_2_) and hydrogen peroxide (H_2_O_2_) as well as hydroxyl radical (OH^•^) and superoxide anion (O_2_^−^) [62,63]. The ROS exert their functions, which can be either beneficial roles or toxic roles depending on their concentration. At low concentration, ROS functions as signaling molecules that regulate various physiological processes via ROS-mediated signaling transduction, whereas at high concentrations, they can cause cellular damage as well as programmed cell death (PCD) [64,65]. In plants, the ROS are derived from the by-products of diverse metabolic pathway and the cellular compartments, including mitochondrial complex I and III in mitochondria, photosystems I and II in chloroplasts, peroxisome, and NADPH oxidase in plasma membrane during the response to stress conditions [17,66,67,68]. Imbalances and surplus of the ROS productions lead to membrane of lipid peroxidation and protein oxidation, as well as damage to a variety of macromolecules, including cell structures, respiratory and photosynthetic complex, and nucleic acids, suggesting that the cellular homeostasis of ROS levels is crucial for normal growth and development as well as LT stress response in plants [17,69].

**Figure 1:**
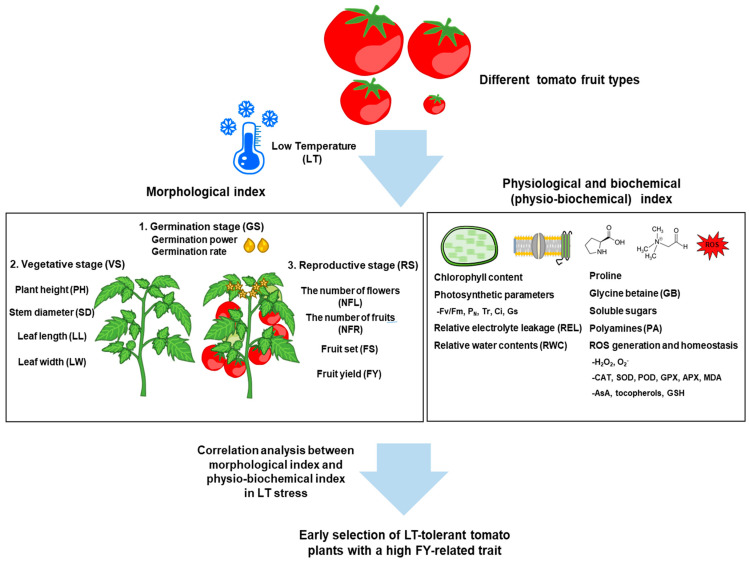
Schematic representation of the factors involved in morphological, physiological, and biochemical changes in tomato plants during the vegetative and reproductive growth stages and the strategy for early selecting LT-tolerant tomato plants under low temperature (LT) stress condition depending on tomato fruit types. The correlation analysis between morphological index and physio-biochemical index which includes a wide range of variables with large-scale analysis can be harnessed to establish breeding programs for early selecting LT-tolerant tomato plants together with a high fruit yield (FY) trait. MDA; malondialdehyde, CAT; catalase, SOD; superoxide dismutase, GPX; glutathione peroxidase, APX; ascorbate peroxidase, AsA; ascorbic acid, GSH; glutathione.

Enzymatic and non-enzymatic antioxidant scavenging systems are crucial for the elimination of toxic ROS in plants. Antioxidant enzymes, including catalase (CAT), superoxide dismutase (SOD), glutathione peroxidase (GPX), and ascorbate peroxidase (APX) are essential components for the scavenging system [14]. The antioxidant enzymes are involved in the detoxifying ROS. For instance, SOD catalyzes the conversion of superoxide anion (O_2_^−^) into hydrogen peroxide (H_2_O_2_) via the Fenton reaction, which is subsequently decomposed by POD and CAT into H_2_O and O_2_ [68]. Moreover, APX and GPX utilize ascorbate and glutathione which act as electron donors to decompose H_2_O_2_, respectively [14,63]. Non-enzymatic antioxidants, including compatible solutes, ascorbic acid (vitamin C), tocopherols (vitamin E), and carotenoids carry out ROS detoxification in plants [14,31]. The antioxidant defense systems, including enzymatic and non-enzymatic defense system, for the ROS detoxification play a pivotal role in tomato tolerance and acclimation under LT stress. Previous study has reported that antisense-mediated transgenic tomato (LeGR) lacking chloroplast glutathione reductase showed that a large accumulation of H_2_O_2_ and a sensitivity to chilling stress in LT stress response, which resulted from the decrease in enzymatic activity of SOD, CAT, and POD and non-enzymatic antioxidants of reduced glutathione (GSH) and ascorbate (AsA) compared to wild-type [70]. Recent studies showed the overexpression of LeGPA1 and LeCOR413PM2, which exhibit LT-tolerant phenotype, accumulates less ROS levels, including H_2_O_2_ and O_2_^−^, as well as MDA (malondialdehyde) contents under LT stress. However, the activity and the gene expression of SOD, CAT, and POD were significantly increased comparted to those in the RI lines [32,33]. Moreover, the overexpression of *Brasscia oleracea* genes (BoCRP1) and *Saussurea involucrata* genes (SiFBA5) promoted LT tolerance in tomato plants with similar results to those described above [25,48]. Intriguingly, the rootstock (Holyc) improved the LT tolerance of the cultivated tomato (Scion) with the observation of the decreased ROS and MDA levels, whereas the increased antioxidant enzymatic activity, including SOD, CAT, and POD in Holyc compared to control (Hetero) [37]. In addition to this, the knockdown line of SlREC2 showed the increased ROS levels, which is crucial for LT tolerance via the SlNCED1-mediated ABA accumulation that regulates the gene expression of CBF-pathway [26]. Further exploration will be required to understand the relationship between ABA or PA metabolism and ROS scavenging defense systems, including non-enzymatic antioxidants during the LT stress response in tomato plants. Taken together, considering the reduction of time for selecting high-yielding and LT-tolerant tomato cultivars at early time, it is worth studying the correlation of more diverse variables, including vegetative and reproductive traits as well as the physiological and biochemical index with large-scale analysis, which will be used to establish breeding programs for selecting LT-tolerant tomato plants with high-yielding selection criteria (Figure 1 and Table 1).

## 4. Molecular Mechanisms Underlying the LT Response in Tomato Plants

### 4.1. LT Perception and LT Response

Low temperature (LT) or cold temperature ranging from 0 to 20 °C is a vital factor for optimizing tomato growth and/or yields at various stages including germination, vegetative and reproductive growth stages [8,11,71]. The plasma membrane is primarily involved in the sensing of external and internal temperature changes, leading to the adjustment of membrane fluidity and cytoskeletal rearrangement [72]. Ca^2+^ influx and intracellular Ca^2+^ concentrations are crucial for plant’s perceiving LT stress. In particular, Ca^2+^ ion channels are responsible for Ca^2+^ influx, which is crucial for the initiation of the LT response [73,74,75]. For instance, Arabidopsis AtMCA1 (MID1-complementing activity 1) and AtMCA2 (MID1-complementing activity 2), Ca^2+^-permeable mechanosensitive channels, are involved in cold-induced Ca^2+^ increase and LT tolerance [76]. In addition, chilling tolerance divergence 1 (COLD1) protein is associated with rice G-protein α subunit 1 (RGA1) in plasma membranes as well as the endoplasmic reticulum (ER) to mediate cold-induced intercellular Ca^2+^ influx, which shows a critical role of Ca^2+^ signaling in cold signal transduction [16]. Recent study has reported that the proteins kinase Open stomata 1/SNF1-related protein kinase 2.6 (OST1/SnRK2.6) is involved in the phosphorylation of *Arabidopsis* AtANN1 (ANNEXIN1) which is a LT-triggered Ca^2+^ permeable transporter, which subsequently leads to Ca^2+^ signaling and thereby positively regulates the transcription levels of cold-responsive genes (CORs) [77]. Recent study has determined that the overexpression of LeCOLD1 dramatically promoted the LT tolerance in tomato plants [78] and tomato OST1 and ANNEXIN are essential for the tolerance to abiotic stresses, including drought and/or salt stress, respectively [79,80]. Furthermore, plant CNGCs, non-selective cation-conducting channels localized in plasma membrane, are involved in providing LT response and tolerance via the participation in LT-induced Ca^2+^ influx and Ca^2+^ elevation in the cytosol [81,82,83,84]. In addition to Ca^2+^ influx, the Ca^2+^ efflux is critical role in LT response and signaling. Arabidopsis AtCAX1 (*Arabidopsis thaliana* calcium exchanger I) for a vacuolar membrane Ca^2+^/H^+^ antiporter and GhCAX3 (*Gossypium hirsutum*) for an organellar Ca^2+^ channel are involved in increasing cold-responsive gene expression during the LT-acclimation response [72,77], suggesting that the regulation of Ca^2^ concentrations in plant cells is critical for LT stress response. Although the importance of Ca^2+^ influx and/or efflux channels has been studied in other crops including *Saccharum*, *Oryza sativa*, *Chinese jujube*, and *Gossypium hirsutum* during LT stress, the functional analysis of the LT-involved Ca^2+^ influx and/or efflux channels or transporters in tomato plants is still lacking of sufficient knowledge. Further study is necessary to investigate the functional roles of Ca^2+^ channels or transporters in the response of tomato to LT stress.

### 4.2. LT Signaling Pathways via a Calcium Molecule

Ca^2+^ plays a critical role in a signal transduction during LT sensing and response and the intracellular calcium levels in the cytosol are increased by Ca^2+^ permeable channels [72]. The increased Ca^2+^ levels subsequently activate the calcium-responsive proteins functioning as Ca^2+^ sensors, including calmodulin (CaM), CAM-like proteins (CML), calcium-dependent protein kinases (CDPK), and calcineurin B-like proteins (CBLs), which is essential for LT-induced Ca^2+^ signaling pathways to amply and/or express LT-related genes [72,73]. Previous research has showed that calcineurin B-like protein-interacting protein kinase (CIPK) is associated with CBLs to regulate CIPK activation and target localization in the response of plants to LT stress [72]. Recent studies on several Ca^2+^ sensors in the tomato plant have demonstrated that the overexpression of SlCML37 enhances LT tolerance in tomato fruit [85], whereas knock-down transgenic lines of SlCaM6, SlCIPK1, and SlCIPK8 result in a significant LT sensitivity compared to wild-type [86,87]. In addition to Ca^2+^ sensors, Arabidopsis calcium/calmodulin-regulated receptor-like kinases 1 (CRLK1), a plasma membrane-associated serine/threonine kinase, is positively involved in LT stress response [16,72]. The CRLK1 is associated with MEKK1 to activate the mitogen-activated protein kinase (MAPK) cascade signaling pathway, including MAPK, MAP2K (MKK or MEK), and MAP3K (MAPKKK or MEKK), which is crucial for LT stress response [88]. Notably, the components, including Arabidopsis MAP2K and MAP3K are activated by LT stress and the MKK2 signaling pathway has been implicated inducing COR gene expression and promoting LT tolerance in Arabidopsis plants [17]. However, cellular functions including Ca^2+^ sensors and CRLKs remain to be analyzed in tomato plants. It will be a great effort to further explore an in-depth molecular link between the Ca^2+^ sensors and CRLKs and MAPK cascade signaling pathway in the response of tomato to LT stress.

### 4.3. LT Signaling Pathways via ROS Molecules

ROS molecules in plants can act as a key molecule to transmit its signal to downstream machinery [63,68]. ROS signaling can be generated from cell wall and apoplast, cytosol and nucleus, and organelles such as peroxisomes, chloroplasts, and mitochondria indicating that these different pathways can play a critical role in LT stress response. ROS are involved in plant stress signaling via oxidative post-translational modifications (Oxi-PTMs) that leads to the conformational changes of target proteins, which further regulate their activity or subcellular localization [68]. The previous study has shown that Frostbite1 (FRO1) encoding the Fe-S subunit of the mitochondrial complex I, regulates the accumulation of ROS in the leaves under LT stress [89]. Moreover, CHY1 encoding a peroxisomal β-hydroxyisobutyryl-CoA hydrolase in plant is involved in the ROS generation, which plays an important role in LT tolerance [90]. Recent study in tomato GLR3. 3 and GLR3. 5, γ-glutamylcysteine synthetases, has demonstrated in a crucial role in LT acclimation-induced cold tolerance via the regulation of apoplastic H_2_O_2_ production and redox balance in tomato plants [91].

The ROS signaling is associated with the MAPK cascade signaling to alter gene expression [92]. *Arabidopsis* MPK3, MPK4, and MPK6 are modulated via the involvement of the ROS under diverse abiotic stresses [93]. Remarkably, the AtMPK6 activity is promoted by osmotic and LT stress [94]. In tomato, previous study has reported that H_2_O_2_ as well as LT induce SlMPK7 expression and the overexpressed transgenic plants of SlMPK7 exhibits LT tolerance [95]. Moreover, the treatment of H_2_O_2_ is involved in the gene expression of SlMAPK1/2/3, which contribute to LT tolerance in tomato plants, implying that the ROS are crucial for SlMAPK cascade signaling for gene expression during LT [96]. It will be next potentially interesting to study understanding the in-depth cellular mechanisms of the ROS-induced Oxi-PTM as well as ROS-involved MAPK cascade signaling pathway in the response of tomato to LT stress.

### 4.4. The LT Signaling Transduction via a CBF Dependent Pathway

A cold signal is transduced downstream to reprogram the expression of cold-responsive genes via either CBF-dependent or CBF-independent pathway, which is a key mechanism for acclimating or coping with LT stress in plants [97]. The C-repeat binding factor (CBF) signaling pathway, also known as dehydration-responsive element binding protein 1 (DREB1), is associated with a series of molecular events that induce the activation of genes responsible for promoting LT tolerance [98]. The CBF genes (CBFs) encode APETALA2/ethylene-responsive element binding factor (AP2/ERF)-type transcriptional factors and are rapidly accumulated during the early LT response (<15 min) and exhibit the maximum expression after the 2 h of LT exposure [99,100]. In particular, the CBFs can bind to the conversed C-repeat/dehydration response element (CRT/DRE) motifs in the promoter regions of CORs, which influences the positive regulation of the gene expression [11]. Importantly, CBFs are redundant in the regulation of CORs expression during LT stress and the overexpression of CORs contributes to LT tolerance in plants [101,102]. Previous studies have showed that the overexpression of *Arabidopsis* CBF1 regulates the positive expression of *COR* genes, which confers LT adaptations [98]. CBF2 functions as a negative regulator of the CBF1 and 3 gene expression and the functional analysis has shown that the CBF2 mutant plant increased LT tolerance [103]. Moreover, the functional genomic studies of *cbf* double and triple mutant plants have demonstrated that the CBFs play a pivotal role in LT tolerance [101,104]. In addition to this, CaM-binding transcription activators (CAMTAs) are involved in a positive regulation of CBF expression [105,106]. A previous study has clearly determined that CAMTA2 is associated with CGCG-box in *Arabidopsis* CBF2 promoter regions [107]. CAMTA3 and 5 participate in a rapid temperature reduction, which results in the induction of CBF1 gene expression [108]. Notably, a recent study in tomato has revealed that *slcbf1* mutant using the clustered regularly interspaced short palindromic repeats (CRISPR)-associated protein-9 nuclease (Cas9) system showed severe chilling sensitivity phenotype compared to wild-type [109]. Furthermore, ethylene (ET) biosynthesis or ET signaling-involved SlCBF1 gene expression conferred LT tolerance in tomato fruit [110]. The coordination of HY5 and MYB15 mediates the expression CBF1-3 genes resulted in LT tolerance in tomato plants [111], suggesting that the regulation of CBFs gene expression in tomato plants also plays a crucial role in LT tolerance.

The CBFs are modulated by inducer of the CBF expression (ICE), classified as a MYC-type bHLH transcription factor in the response of plants to LT [112]. In detail, the ICE functions as an upstream transcription factor and is involved in a positive CBFs expressions by associating with cis-element of MYC (CANNTG) in the CBF promoters during LT stress response [112,113]. Previous studies have reported the *ice1* mutant plant influence not only the gene expression of CBF3, but also many downstream CORs in LT conditions [114] and the overexpressed ICE1 plant affected CBF gene regulation [31,115]. In addition, LT-induced OST1 phosphorylates ICE1, enhancing its stabilization via SAP and Miz1 (SIZ1)-involved in sumoylation, whereas ICE1 is degraded by ubiquitination [116]. High expression of osmotically responsive gene 1 (HOS1), RING E3 ubiquitin ligase in the nucleus, is involved in a negative regulator of ICE1 [117]. Previous study has exhibited that the expression of LT-induced genes, including CBFs and CORs, is downregulated in the HOS1-overexpression plants during LT stress response [118] and a study in tomato plants has clearly showed that SlICE1a also plays a key role for LT tolerance and other abiotic tolerance in transgenic tobacco plants, similar to *Arabidopsis* plants [39].

MPK6 harbors the ability to phosphorylate MYB15, which reduces the affinity of MYB15 to bind to the CBF3 promoter regions and diminishes LT tolerance in plants [119]. The PUB25 and PUB26 harboring E3 ligase activity enable to associate with MYB15 TF in *Arabidopsis*, impairing its DNA-binding ability, which result in a positive regulation of CBF gene expression [120]. Interestingly, a recent study has shown that BYPASS1-LIKE (B1L), which localize in both nucleus and cytoplasm in *Arabidopsis*, enhances the CBF stability via interaction with 14-3-3 protein that is phosphorylated by cytoplasmic receptor-like kinase 1 (CRPK1) protein and affects CBF destabilization during LT stress response [121]. Moreover, the activation of *Arabidopsis* MKK2 resulted in the positive regulation of CBF2 and 3 during LT stress response [122], whereas MPK3 as well as MPK6 is involved in the destabilization of the ICE1 protein via phosphorylation, which hinders the expression of CBFs, leading to LT sensitivity [123]. Remarkably, a recent study has shown in tomato plants that SlMPK1 and SlMPK2 are involved in the SlBBX17 phosphorylation, which promotes the complex of SlHY5 and SlBBX17 that subsequently regulates SlCBFs to confer LT tolerance [124]. Taken together, LT signaling transduction via CBF dependent pathway plays a significant role in enhancing LT tolerance in tomato plants by regulating COR genes via the TFs. Further studies are needed to elucidate how the precise mechanisms of how other key modulators including CAMTAs, MYBs, and MAPKs are involved in CBF-dependent pathway to fine-tune their expression in tomato plants during LT stress response.

### 4.5. The LT Signaling Transduction via a CBF-Independent Pathway

Although the regulation of COR genes is primarily essential for improving LT tolerance during LT stress response and LT acclimation, previous and current transcriptomic analyses have proved that CBFs only affect the regulation of approximately 10 to 20 percent of COR genes, indicating that the COR genes also confers LT tolerance via CBF-independent signaling pathways [101,112,125]. For instance, eskimo1 (*esk1*) mutant plant in *Arabidopsis* confers continuous LT tolerance by the increased accumulation of free proline levels [126]. A recent study has shown that heat shock transcription factor A1d (HSFA1d) plays an important role in hypocotyl elongation in the response of the plants to LT stress via the association with the promoter regions of RPL9 and RPL18 ribosomal proteins, which confer LT tolerance [127]. The overexpression of heat shock transcription factor C1 (HSFC1) in *Arabidopsis* plant is involved in the positive expression of COR genes, enhancing LT tolerance [128]. Moreover, *Arabidopsis* HOS9 conferred LT tolerance without altering the gene expression of CBF1-3 [129]. It has been reported that more than 10 transcriptional factors, including heat shock transcription factor C1 (HSFC1), Related to ABI3/VP1 (RAV1), Elongated hypocotyl 5 (HY5), Ethylene-responsive element-binding factor 5 (ERF5), MYB44/73, Zinc finger of *Arabidopsis thaliana* transcription factor 10 (STZ/ZAT10), and Zinc finger of *Arabidopsis thaliana* transcription factor 12 (ZAT12) regulate the expression of CORs genes via a CBF-independent pathway during LT stress response [130,131,132]. Intriguingly, recent studies have in tomato plants showed that the overexpression of CsPIF8 also confers LT tolerance in *S. lycopersicum* [133]. SlNAC3 affects the transcription of ET biosynthesis-related genes and the knockdown transgenic plants are LT tolerant compared to wild-type [134], implying that diverse CBF-independent LT signaling pathways play a pivotal role in the response of the plants to LT stress. Further exploration will be necessary to fully elucidate the in-depth fundamental molecular and regulatory mechanisms underlying post-transcriptional, post-translational, and hormonal modifications as well as metabolic changes in tomato plants during LT response in CBF-independent pathways.

### 4.6. The Cellular Roles of RNA-Binding Proteins in LT Response

Posttranscriptional RNA metabolism in nucleus and organelles (chloroplasts and mitochondria), including intron splicing, RNA stability and export, and translation control, is a potent regulatory mechanism of plant growth, development, and stress responses [135]. Diverse RNA-binding proteins (RBPs) harboring different motifs or domains, including RNA-recognition motif (RRM), K-homology domain, zinc-finger motif, cold-shock domain (CSD), glycine-rich domain, DEAD-box motif, pentatricopetide repeat proteins (PPRs), and chloroplast RNA splicing and ribosome maturation domain (CRM), are crucial cellular modulators regulating stress responses in plants [135,136,137]. The roles of RBPs in LT response have been demonstrated in diverse plant species, including Arabidopsis, rice, wheat, maize, and rape [137]. In particular, glycine-rich RNA-binding proteins (GR-RBPs) harboring glycine-rich domain in combination with RRM, CSD, or zinc finger motif play integral roles in regulating stress response in crops [138].

**Figure 2:**
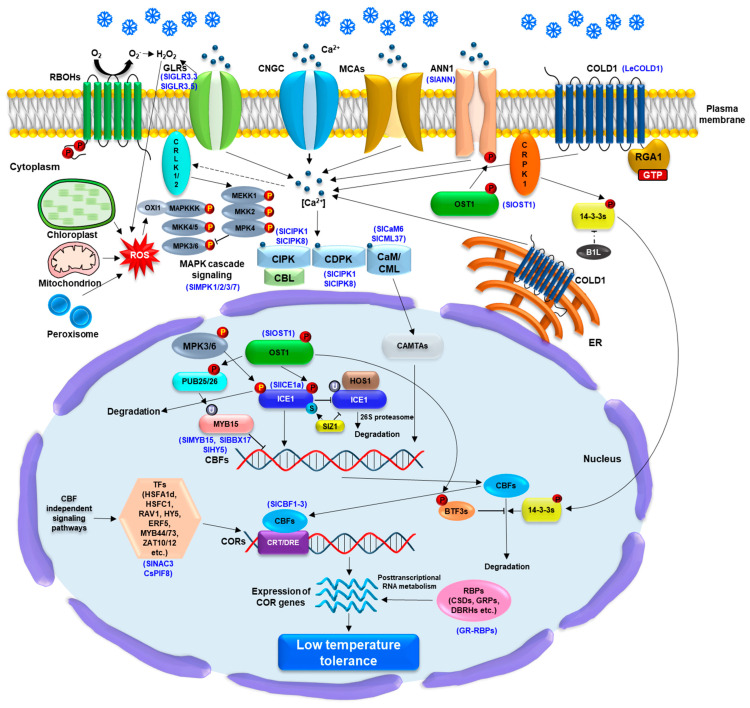
Overview of low temperature (LT) sensing, signaling transduction, and gene regulation via CBF-dependent or CBF–independent signaling pathway in planta. LT is considered to be sensed by the plasma membrane (PM)-and endoplasmic reticulum (ER) membrane-localized COLD1 (chilling tolerance divergence 1) with the interaction of rice G-protein subunit 1 (RGA1), which lead to Ca^2+^ influx into the cytoplasm. Arabidopsis Ca^2+^ channels, CNGCs, MCA1/2 (MID1-complementing activity 1/2), glutamate receptor-like channels (GLRs), and the Ca^2+^ transporter AtANN1 (ANNEXIN1) also play a crucial role in Ca^2+^ influx into the cytoplasm, which subsequently activates Ca^2+^ sensors such as calmodulin (CaM), CAM-like proteins (CML), calcium-dependent protein kinases (CDPK), and calcineurin B-like proteins (CBLs). The activation of CaM/CML induces CAMTAs activity could affect a positive regulation of CBF (C-repeat binding factor) expression. The phosphorylation of LT-activated OST1 (Open stomata 1) is important for AtANN1 activation as well as CBF-dependent signaling pathway. The phosphorylation of ICE1 (inducer of the CBF expression 1) is regulated by LT-activated OST1 and MKK4/5-MPK3/6 cascades, which enhance and diminish the protein stability, respectively, during LT stress response. The interaction of OST1-ICE1 protein complex hinders the E3 ligase high expression of osmotically responsive gene 1 (HOS1)-associated the ICE1 degradation via 26S proteasome system. The SUMO E3 ligase SAP and Miz 1 (SIZ1)-associated ICE1 sumoylation promotes the ICE1 stability. The phosphorylation of the U-box E3 ligases PUB25 and PUB26 by the OST1 is essential for regulating the degradation of the MYB15, a negative regulator of CBFs upstream. Moreover, LT-activated PM receptor-like cytoplasmic calcium/calmodulin-regulated receptor-like kinases 1/2 (CRLK1/2) and cytoplasmic receptor-like kinase 1 (CRPK1) enable to induce the phosphorylation of MEKK1 and 14-3-3 protein which negatively affect the stability of ICE1 and CBFs, respectively, whereas the BTF3s and B1L are involved in the positive stability of CBFs. SlMPK1 and SlMPK2 in tomato plants are involved in the SlBBX17 phosphorylation, which regulates SlCBFs and confers LT tolerance. The ROS generation and redox balance from chloroplast, mitochondria, peroxisome, and apoplast, including respiratory burst oxidase homologues (RBOHs) and tomato GLR 3.3/3.5 also affect CBF-dependent signaling via ROS-mediated OXI1-MAPKKK cascade under LT stress response. Aformentioned LT-induced accumulation of CBFs lead to upregulation of cold-responsive (COR) genes via binding to C-repeat/dehydration response element (CRT/DRE) motif in the promoters of CORs genes. In CBF-independent signaling pathway, transcription factor (TF), including AtHSFA1d, AtRAV1, AtHSFC1, AtHY5, AtERF5, AtZAT10/12, MYB44/73, CsPIF8, and SlNAC3 in tomato, as well as RNA-binding proteins (RBPs) play a pivotal role in the transcriptional and post-transcriptional regulation of the COR genes, which confer LT tolerance to plants. Blue letters in brackets indicate that genes studied in tomato plants.

The significance of RBPs, particularly GR-RBPs, in the development and ripening of tomatoes under normal conditions and LT response is recently emerging. For instance, SlORRM4, GR-RBP 5 in tomatoes, is associated with fruit ripening by modulating RNA editing in chloroplasts and mitochondria [139,140]. In addition, RZ1AL, a zinc-finger GR-RBP, participates in regulating carotenoid biosynthesis and tomato fruit ripening under normal conditions [141]. A previous report has demonstrated that overexpression of LeRBP1, a GR-RBP in tomato, increases total protein contents of tomato fruits under postharvest cold-storage conditions [142]. A recent genome-wide analysis revealed that the tomato genome encodes eight GR-RBPs and most of the GR-RBPs genes are upregulated during cold stress [143]. It will be of worth to further explore whether GR-RBPs and other RBPs play a significant role in LT response in tomatoes as observed in other crops. Overall, the pathways including LT perception and response, signaling transduction, and gene regulation via CBF-dependent and independent pathways are described in Figure 2.

### 4.7. Epigenetic Regulation of Fruit Ripening and Abiotic Stress in Tomato Plants

#### 4.7.1. DNA Methylation in Fruit Ripening and Abiotic Stress Response

Recent advance in epigenetics increases our understanding of the pivotal role of epigenetic regulators, including DNA methylation, histone modifications, and noncoding RNAs (ncRNAs), in tomato fruit development, ripening, and stress responses [144,145]. DNA methylation and demethylation, mainly occurring at 5-methylcytosine, is closely associated with tomato fruit ripening [146]: the global DNA methylation level decreases as the tomato fruits mature [147] and SlDML2, DNA demethylase 2 in tomatoes, is necessary for DNA demethylation during ripening [148]. Moreover, DNA methylation regulates the pigment accumulation, flavor metabolism, and texture of tomato fruits [146].

The role of DNA methylation in LT response in tomatoes is emerging. For instance, chilling stress inhibits *SlDML2* expression, which suppresses DNA demethylation and ripening [149]. In addition, chilling stress-mediated changes in DNA methylation levels in tomato fruits are associated with flavor loss and variation in the transcriptional levels of key ripening genes [150]. Notably, a recent comparative analysis of the methylome and transcriptome of tomato fruits during postharvest storage at LT revealed that postharvest ripening at LT is closely associated with the DNA methylation-mediated gene regulation [151]. However, it remains to be determined how DNA methylation on specific ripening-related genes is regulated at different developmental and ripening stages of tomatoes under chilling stress and which DNA methyltransferases and demethylases are associated with the altered methylation levels in tomatoes under abiotic stresses.

#### 4.7.2. Histone Modifications in Fruit Ripening and Abiotic Stress Response

Histone modifications, including methylation and acetylation at lysine residues, are potent regulatory mechanisms of tomato fruit development and ripening. SlLHP1b, a polycomb group protein regulating histone methylation in tomatoes, represses fruit ripening by modulating H3K27 methylation [152]. Tomato jumonji domain-containing protein 6 (SlJMJ6), a histone demethylase, promotes tomato fruit ripening by mediating H3K27me3 demethylation in several ripening-related genes [153]. Moreover, a recent study demonstrated that SlJMJ3 accelerates tomato fruit ripening by modulating the expression of multiple ripening-related genes involved in ethylene response, carotenoid metabolism, cell wall modification, and DNA methylation [154]. Notably, SlJMJ7, an H3K4 demethylase, was shown to act as a master negative regulator of fruit ripening not only through direct removal of H3K4me3 from multiple ripening-related genes, but also through crosstalk between histone and DNA demethylation [155]. Acetylation and deacetylation of histone tails, which are catalyzed by histone acetyltransferase (HAT) and histone deacetylase (HDAC), respectively, is another potent epigenetic mechanism governing tomato fruit ripening. The SlHDA1 and SlHDA3 were shown to delay the ripening process and carotenoid accumulation of tomatoes [156,157]. SlHDT1, a HDAC gene in tomatoes, is a negative regulator controlling ethylene and carotenoid biosynthesis during fruit ripening [158].

In addition to the crucial role of histone modifications in tomato fruit ripening under normal conditions, the significance of histone modifications in abiotic stress responses is also emerging. Recent studies have demonstrated that RNAi-mediated silencing of *SlHDA1* or *SlHDA3* resulted in poorer shoot and root growth, earlier yellowing, and faster degradation of chlorophyll compared to wild-type under drought or salt stress [159,160], emphasizing the critical role of histone deacetylation in safeguarding tomato plants against drought and salt stress. However, it remains to be discovered whether histone methylation and acetylation are also involved in LT response in tomatoes and which histone modifiers, including histone methyltransferases and HATs, are associated with the altered histone methylation and acetylation levels in tomatoes under abiotic stresses.

#### 4.7.3. Noncoding RNAs in Fruit Ripening and Abiotic Stress Response

In addition to DNA methylation and histone modifications, ncRNAs, including microRNA (miRNA), long noncoding RNA (lncRNA), and circular RNA (circRNA), are another epigenetic factor regulating tomato fruit ripening [161]. A recent study has revealed that loss of function of *SlMIR164A* results in accelerated fruit ripening and enhanced chloroplast development by targeting *SlNAM2* and *SlNAM3* [162]. Moreover, the miR164a-NAM3 module confers cold tolerance in tomato plants via regulating *SlACS1A*, *SlACS1B*, *SlACO1*, and *SlACO4* expression to induce ethylene synthesis [163]. Notably, miR162 negatively regulates stomatal opening and photosynthesis activity via ABA signaling pathway in tomato plants in response to low night temperature [164]. Through deep sequencing, a total of 1018 circRNAs were identified in tomato fruits, some of which are associated with pigment synthesis [165] and several lncRNAs involved in ethylene biosynthesis and signaling, fruit flavor, and ripening were identified in tomatoes [166]. Noticeably, deep sequencing and bioinformatics analysis revealed 239 lncRNAs possibly involved in chilling injury in tomato fruits [167]. It will be interesting to further explore whether miRNAs, lncRNAs, and circRNAs are associated with LT response in tomatoes and to determine the regulatory mechanisms underlying the ncRNA-mediated control of the development and ripening of tomato fruits in response to LT stress.

### 4.8. RNA Methylation in Fruit Ripening and Abiotic Stress Response

Chemical modification in RNAs is a potent epigenetic process affecting entire growth and development of plants. Among over 160 chemical modifications identified in RNAs, N6-methyladenosine (m^6^A) is the most about modification present in eukaryotic mRNAs, which plays a crucial role in plant growth, development, and stress responses [168,169]. A recent transcriptome-wide analysis of m^6^A methylomes identified a large numbers of m^6^A-modified genes involved in the expansion and ripening of tomato fruits [170]. The m^6^A marks are added, removed, and decoded by m^6^A writers, erasers, and readers, respectively [18,169]. A recent genome-wide analysis identified the m^6^A writers, erasers, and readers in tomatoes and revealed their expression patterns under various abiotic stresses [171]. Notably, disruption of SlALKBH2, an m^6^A eraser in tomato, was associated with delayed tomato fruit ripening by increasing the stability of *SlDML2* transcripts [172]. SlYTH1, an m^6^A reader in tomato, affects the growth and fruit shape of tomatoes by regulating gibberellin biosynthesis [173]. These studies point to the crucial role of m^6^A modification in the vegetative growth and the expansion and ripening of tomato fruits.

The impact of m^6^A on stress response in tomatoes is recently emerging. Through nanopore direct RNA sequencing, overall m^6^A patterns and the m^6^A-modified genes potentially involved in tomato fruit chilling injury have been determined [174]. In addition, many m^6^A-modified genes related to lipid metabolism, ATPase activity, and ABA biosynthesis were altered in tomato anthers under LT, suggesting a molecular link between m^6^A methylation and tomato anther development under LT stress [175]. Interestingly, overexpression of SlYTP8 increased the sensitivity of tomato plants to LT stress, whereas overexpression of SlYTP9 increased the resistance of tomatoes to waterlogging stress. These studies clearly demonstrate that m^6^A modification plays a crucial role in the response of tomatoes to abiotic stresses. However, it remains to be discovered how m^6^A writers, erasers, and readers are regulated by LT stress and what are the target genes modulated by m^6^A writers, erasers, and readers, which eventually influences LT response in tomatoes. Moreover, considering that the SlALKBH2-SlDML2 module is involved in tomato fruit ripening regulation [172] and a crosstalk exists between RNA methylation and epigenetic regulators [18], it will be of great interest to further explore a molecular link between m^6^A RNA methylation and epigenetic regulators in LT response in tomatoes.

## 5. Conclusions and Future Prospects

Recent studies in tomato plants have elucidated that LT stress adversely affects vegetative parameters of LL, LW, and SD, and reproductive parameters of NFR, NF, FS, and FY. Moreover, LT stress leads to significant physiological and biochemical changes, including chlorophyll contents, photosynthetic parameters, REC, RWC, osmolytes (proline, soluble sugar, and glycine betaine), PAs, ROS, and antioxidants during the vegetative and developmental growth stages. To increase the efficiency for rapid selection of LT-tolerant tomato plants and to breed high-yield and high-quality tomato varieties, the correlation study of more diverse variables associated with vegetative and reproductive traits, as well as physiological and biochemical index remains to be explored. Moreover, recent studies have determined molecular mechanism underlying LT-mediated COR gene regulation and epigenetic regulation in tomato plants. The coordination of LT perception, including Ca^2+^ influx/efflux channels and LT signaling transduction via ICE-CBF-dependent or CBF-independent pathways, MAPK cascade, Ca^2+^ molecules, and ROS molecules, orchestrates the expression of COR genes in tomato response to LT stress. Moreover, crosstalk between epigenetic regulators and RNA m^6^A modification is emerging in tomato plants, which will be pivotal for fine-tune regulation of the transcripts associated with the vegetative growth and fruit ripening of tomatoes during LT stress response. In-depth transcriptional regulatory networks involving CBF genes, signaling pathways, interactions of various TFs and regulatory modulators, and epigenetic regulators remain to be explored in tomato plants.

The identification and functional genomic study of the genes associated with LT stress response and tolerance have advanced our understanding of the molecular mechanism underlying LT response in tomato plants. However, it is still not sufficient to produce the LT-tolerant tomato cultivars harboring desired traits via current molecular breeding or genetic biotechnology. Given that traits associated with LT tolerance exhibit quantitative inheritance, to enhance the efficiency of selecting LT-tolerant tomato lines, it is indispensable to develop molecular marker via a marker-assisted selection system (MAS), genotyping-by-sequencing (GBS), and GWAS (genome-wide association studies) for candidate genes, and bi-parental QTL (quantitative trait locus) mapping. Moreover, advanced genome editing techniques, including the CRISPR/Cas9 system and CRISPR/Cas13 systems, can be employed in conjunction with molecular breeding to introduce beneficial genes and neutralize harmful genes for the development of LT-tolerant tomato elite lines. The integration of comprehensive physiological and molecular understanding of LT stress response and powerful genome editing tools will accelerate the breeding of LT-tolerant tomato varieties. It is challenging to engineer cold-tolerant crops, and we anticipate novel development in coming years.

## Figures and Tables

**Table 1:** Physiological and biochemical changes in the response of tomato to LT stress.

Index	Gene Name/Treatment	Physiological and Biochemical Changes	Ref.
Chlorophyll	SiFBA5	Increase in the overexpression plant Enhanced LT tolerance	[25]
Photosynthetic parameters (*Fv/Fm*, *P_N_*, *Ci*, *Tr*, *Gs*)	SiFBA5 SlREC2	Decease in the sensitive and mutant plants Sensitive to LT stress	[24,25,26]
REL	LeGPA1 LeCOR413PM2 ShPP2-1 SlREC2	Increase in the RNAi transgenic plants Decrease in the overexpression plants Enhanced LT tolerance	[11,26,32,33]
RWC	LEGPA1 LECOR413PM2 Rootstocks (Holyc)	Increase in the overexpression plants and rootstocks Enhanced LT tolerance	[32,33,37]
Proline	Exogenous treatment	Enhanced LT tolerance	[40]
	Osmotin	Increase in the transgenic plant Enhanced LT tolerance	[42]
	*high pigment 1* LeGPA1 LeCOR413PM2 BOCRR1	Increase in the mutant and overexpression plants Enhanced LT tolerance	[32,33,47,48]
GB	Exogenous treatment	Enhanced LT tolerance	[43,44,45]
	BADH	Increase in the overexpression plant Enhanced LT tolerance	[46]
	*high pigment 1*	Increase in the mutant Enhanced LT tolerance	[47]
Soluble sugars	LeGPA1 LeCOR413PM2 BOCRR1	Increase in the overexpression plant Enhanced LT tolerance	[32,33,48]
Put	SIMYC2	Decreases in the RNAi transgenic plant Sensitive to LT stress	[61]
Spd	ShWRKY55	Increases in the LT-exposed LA1777 tomato via ShWRKY55 and ShSAMDC2 regulation Enhanced LT tolerance	[53]
ROS	LeGR	Increase in antisense transgenic plants Sensitive to LT stress	[70]
	LeGPA1 LeCOR413PM2 BoCRP1 SiFBA5 Rootstock (Holyc) SlREC2	Decrease in the overexpression plants and rootstock Enhanced LT tolerance	[25,32,33,37,47,48,70]
CAT, SOD, POD	LeGPA1 LeCOR413PM2 BoCRP1 SiFBA5 Rootstock (Holyc) SlREC2	Increase in the overexpression plants and rootstock Enhanced LT tolerance	
APX	BoCRP1	Increase in the overexpression plant Enhanced LT tolerance	[48]
GSH, AsA	LeGR	Decrease in antisense transgenic plant Sensitive to LT stress	[70]

## Data Availability

All data are included in the paper.
